# Clean production and utilisation of hydrogen in molten salts

**DOI:** 10.1039/d0ra06575g

**Published:** 2020-10-01

**Authors:** Ali Reza Kamali

**Affiliations:** Energy and Environmental Materials Research Centre (E^2^MC), School of Metallurgy, Northeastern University Shenyang 110819 People's Republic of China ali@smm.neu.edu.cn a.r.kamali@cantab.net

## Abstract

Green and low cost production of strategic materials such as steel and graphene at large scale is a critical step towards sustainable industrial developments. Hydrogen is a green fuel for the future, and a key element for the clean production of steel. However, the sustainable and economic production of hydrogen is a barrier towards its large scale utilisation in iron and steelmaking, and other possible applications. As a key challenge, the water electrolysis, which is commonly used for the carbon-free production of hydrogen, is uneconomic and involves various problems including the corrosion of equipment, the use of expensive catalysts and high over-potentials, limiting its viability. Moreover, the hydrogen transportation from the electrolyser to the utilisation unit is problematic in terms of cost and safety. From a thermodynamic point of view, the potential and efficiency of the water splitting process can greatly be improved at high temperatures. Therefore, a practical approach to resolve the above-mentioned shortcomings can be based on the electro-generation of hydrogen in high temperature molten salts, and the utilisation of the generated hydrogen *in situ* to produce metals, alloys or other commercially valuable materials. Clean production of alloy powders is particularly interesting due to the rising of advanced manufacturing methods like additive manufacturing. The hydrogen produced in molten salts can also be used for the large scale preparation of high value advanced carbon nanostructures such as single and multi-layer high quality graphene and nanodiamonds. The combination of these findings can lead to the fabrication of hybrid structures with interesting energy and environmental applications. Surprisingly, the production of a large variety of materials such as Fe, Mo, W, Ni and Co-based alloys should be achievable by the electrolytic hydrogen produced in molten salts at a potential of around 1 V, which can easily be powered by advanced photovoltaic cells. This review discusses the recent advancements on these topics.

## Abstract 2

Green and low-cost electro-generation of hydrogen in molten salts provides unique opportunities for the sustainable production of a range of advanced materials from high quality graphene to metal/alloy powders.

## Graphical abstract

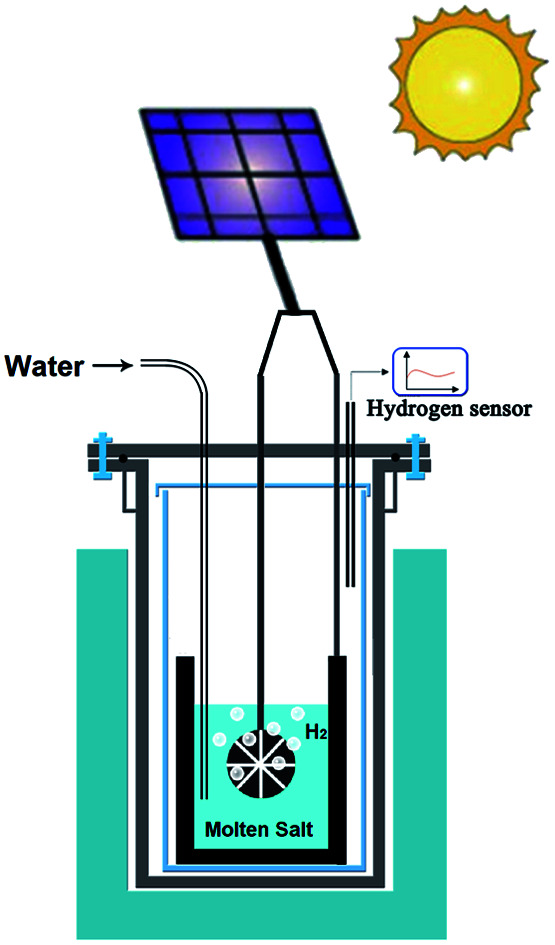

## 1. Hydrogen and sustainability

Hydrogen has been playing an increasingly important role in materials processing and technological developments, such as the upgrading of petroleum hydrocarbons including crude oil, gasoline and diesel with a capacity of over 100 million barrels per day. Hydrogen is also one of the main ingredients in the production of methanol and ammonia with an annual production of over 175 million tons. It is also used as the shielding gas in welding and the coolant in power stations. Apart from these traditional applications, hydrogen is considered the most environmentally attractive energy carrier with the highest gravimetric energy density of all known substances,^1–3^ around 120 MJ kg^−1^,^4^ which is considerably greater than those of gasoline (∼45 MJ kg^−1^), methane (∼50 MJ kg^−1^) and Li-ion batteries (0.9–3.6 MJ kg^−1^).^5^ Furthermore, the combustion of hydrogen is emission-free as it produces only water. Hydrogen is also a green fuel for the production of electricity using a variety of developed fuel cell systems^6^ with water as the by-product, applicable in a wide range of applications from microelectronics and portable devices^7^ to transportation^8,9^ and large scale power generation sectors.^10,11^

Hydrogen is also considered as a green reducing agent for the sustainable production of a large variety of metals such as molybdenum,^12,13^ nickel,^14^ germanium,^15^ tungsten,^16^ and cobalt^17^ as well as alloys such Fe–Mo.^18^ On important possible application of hydrogen is the clean reduction of iron oxides either from oxidised alloys,^19^ or iron ores^20–22^ at 600–800 °C. This application is particularly attractive due to the fact that the steel industry is at the heart of global development with a world production of over 1.8 billion tons, producing more than 3.2 billion tons of CO_2_ every year.^23^

## 2. Global challenges associated with iron and steel production

Iron and steel industry is the responsible for approximately 7% of global CO_2_ emissions.^24^ The 2015 Paris Agreement on climate change implies that this sector must reach zero emissions by 2060–2080.^25,26^ China, with half of the world's steel production, is committed to prevent its CO_2_ emissions from increasing by 2030. To meet this requirement, fundamental technological innovations^27^ should be in place. Currently, steel is commercially produced using two technologies of (a) the integrated blast furnace–basic oxygen furnace (BF–BOF)^28^ or (b) the direct reduced iron–electric arc furnace (DRI–EAF).^29,30^ Blast furnaces use iron ore, scrap metal and coke to produce impure molten iron for conversion in the BOF, along with CO_2_ emissions. Moreover, apart from CO_2_, other hazardous emissions such as SO_2_ and NO_*x*_ are also formed from the oxidation of sulphur, nitrogen and other impurities in coal and coke with negative consequences on human health.^31^ Diminishing the resources of high quality metallurgical coke^32^ as well as associated environmental problems^33^ have been the driving force for the development of alternative technologies such as DRI–EAF, in which natural gas consisting primarily of methane (CH_4_) is often used as the reducing agent, leading to the reduction of emissions to around 0.77–0.92 ton of CO_2_ per ton of produced DRI.^34^ Despite this improvement, in 2018, only around 100 million tons of direct reduced iron (less than 6% of the world primary steel production) was produced world-wide, due to the limited availability of natural gas, its negative environmental impacts and the transportation problems.

Moreover, the natural gas resources mostly contain impurities such as H_2_S and CO_2_, and should be purified before transportation due to the toxicity and corrosion-enhancing characteristics of these compounds. Therefore, the natural gas is difficult to be used in the future sustainable developments.^35–37^ A concept to tackle the CO_2_ emissions is based on the CO_2_ capture.^38,39^ However, its high associated costs, based on the current technologies (US$60–80 per ton of CO_2_), are far more expensive to make this concept commercially viable at large scales.^40^

## 3. Hydrogen for sustainable iron and steelmaking

### 3.1. Opportunities

Hydrogen is an ultimate clean reducing agent that can be employed without producing pollution. Therefore, hydrogen-based production of metals and alloys (especially iron and steel) is of great interest internationally. Several steelmakers have already initiated major projects on the preparation of hydrogen for green steelmaking. These include Green Industrial Hydrogen (GrInHy) conducted by German Salzgitter; H2FUTURE by Austrian voestalpine; and HYBRIT by Swedish entities SSAB (steel manufacturer), LKAB (mining company) and Vattenfall (electricity and heat producers). ArcelorMittal (a Luxembourg-registered steel group) is building a €65 million demonstration plant. Other EU projects include the one conducted by Finish Outotec Circored aiming to use hydrogen for iron making. In China, there are currently more than 260 blast furnaces,^41^ many of which are reaching their end of service life, and can be replaced by clean technologies.

### 3.2. Challenges

Theoretically, production of 1 kg of Fe requires 5.4 × 10^−2^ kg of H_2_, based on the reaction (1).

1 Fe_2_O_3_ + 3H_2_ → 2Fe + 3H_2_O

However, practically, around four times the stoichiometric gas flow is required to support the reduction process.^41,42^ It should be mentioned that hydrogen produced by different methods costs between $1.5 to $6.2 per kilogram.^43^ The lower costs associate with steam methane reforming (SMR) methods that also produce CO_2_ emissions, whilst higher costs (over $2.2 per kilogram) relates to electrolysis approaches, with an efficiency of around 30%.^43,44^ Therefore, the hydrogen required for the clean production of 1 kg Fe would cost around $0.5 which is not economic, considering that the price of producing iron in blast furnace is considerably less expensive.

It should be noticed that, theoretically, for the decomposition of water, a minimum voltage of 1.23 V is required to achieve the cathodic hydrogen and anodic oxygen evolution reactions. Practically, however, the threshold voltage for the water electrolysis is considerably higher at 1.8–2.0 V either in alkaline or acidic electrolytes, due to the presence of ohmic losses, and the activation over-potential caused by electrode kinetics and the sluggish mass transfer at the electrode–electrolyte interfaces.^45–48^ Therefore, a large amount of energy is required to split water (∼4 kW h m^−3^) which is greater than the energy content of produced hydrogen (∼3.5 kW h m^−3^),^49^ making the whole process non-economical on an industrial scale. Other technical problems include the corrosion of the electrodes, containers and compartments;^50^ and expensive catalysts required such as Pt- and Ru-based compounds.^51^ Due to these limitations, the production of hydrogen by water electrolysis accounts for only 4% of the world's hydrogen production.

On the other hand, while the production of hydrogen powered by photovoltaic (PV) panels represents an environmentally preferable way for the green production of hydrogen, this approach is not economic using current technologies. The efficiency (*E*) of such a system comprising the PV panel and the water electrolyser can be expressed by eqn (2):^49^

2 *E* = (*M* × *C*)/(*V* × *I*)

where *M* and *C* are the mass flow rate (g s^−1^), and the higher calorific value of hydrogen (J g^−1^); while *V* and *I* are the voltage (V) and current (A) of the panel, respectively. The overall efficiency of such systems is very low at around 1%.^49^ As realised from eqn (2), to enhance the efficiency, one can reduce the water splitting voltage, achievable by high temperature electrolysis. It is explained in the next section.

## 4. High temperature water electrolysis

**Fig. 1:**
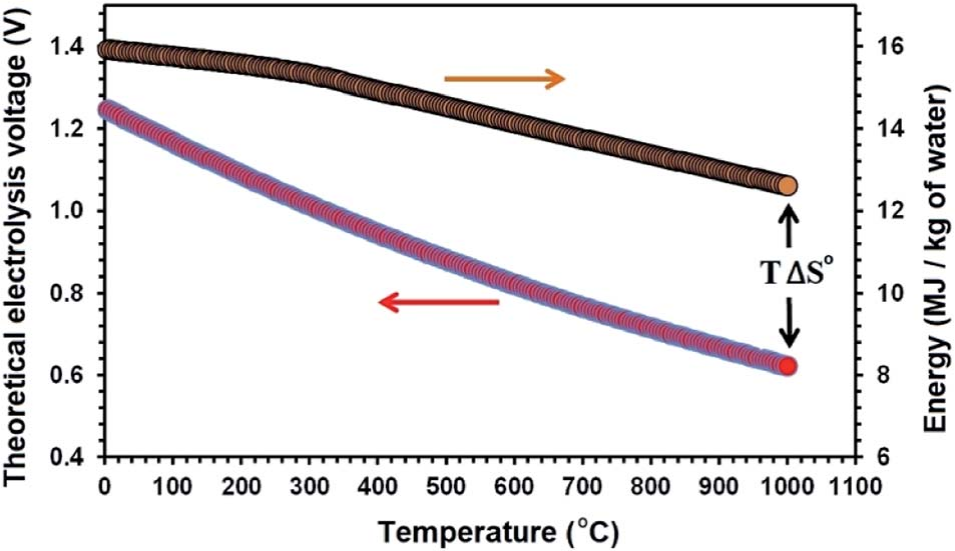
The theoretical electrolysis voltage and the energy required for the electro-decomposition of water into hydrogen and oxygen; H_2_O → H_2_ + 1/2O_2_.

Temperature can influence the energy required for an electrolysis reaction. From the thermodynamic point of view, this can be described by eqn (3):

3 Δ*H*° = Δ*G*° + *T*Δ*S*°

where Δ*H*° is the standard enthalpy change corresponding to the total energy required for the electrolysis process, and Δ*S*° is the associated standard entropy change. *T*Δ*S*° and Δ*G*° are the thermal energy, and the electrical energy required for the electrolysis, respectively. Fig. 1 displays the theoretical voltage and the energy required for the electro-decomposition of water at different temperatures ranging from 0 to 1000 °C.

At present, solid oxide steam electrolysis (SOE) operating at 700–1000 °C is the only way to produce hydrogen at high temperatures,^52,53^ but these systems require expensive materials such as yttria doped zirconia, gadolinia-doped ceria, and lanthanum strontium cobalt ferrite.^54^ Moreover, the SOE electrolysis cells are prone to degrade primarily due to the anode delamination at the interface with the solid electrolyte due to the elevated pressure caused by the electrochemically formed oxygen.^55,56^ Moreover, the transportation of hydrogen from the production unit to the utilisation unit is highly problematic, expensive and dangerous.^57,58^

## 5. Molten salt production of hydrogen from water

As outlined in previous sections, currently there is no technology that can produce hydrogen efficiently, in particular, at high temperatures. A possible approach to achieve this goal can be based on the facile production of hydrogen from water precursor in high temperature molten salts, and the utilisation of the hydrogen produced for the synthesis of interesting materials. This unique methodology is based on the capability of specific molten salt systems to absorb moisture from the environment at high temperatures.^59–62^ For instance, water can be dissolved in molten LiCl to generate hydrogen cations and oxygen anions through a hydrolysis mechanism (reaction (4))^63,64^ considering that the species formed (HCl and Li_2_O) are easily soluble in the melt.^65,66^

4 2LiCl_melt_ + H_2_O_atmosphere_ → 2HCl_[LiCl]_ + Li_2_O_[LiCl]_

Although the standard equilibrium constant of this reaction is low (*K* = 1.05 × 10^−10^ at 700 °C), yet the reaction can proceed at a finite rate by the dissolution of the reaction products in the melt. It should be mentioned that the formation of Li_2_O in the melt is highly beneficial for the fast production of Li-containing ceramic materials such as LiNbO_3_,^64^ Li_2_Fe_3_O_5_,^67^ and Li_2_TiO_3_ (ref. 68) as well as Li_2_CO_3_.^69,70^

Under this situation, the cations present in the molten salt (H^+^ or Li^+^) may be discharged on the cathode under the influence of a cathodic potential applied. In LiCl, possible electrochemical reactions at 660 °C can be described as below:

5 2LiCl → 2Li + Cl_2_(g), Δ*G*° = 666.3 kJ per mole, *E*° = −3.4 V

6 2Li_2_O → 4Li + O_2_(g), Δ*G*° = 948.7 kJ per mole, *E*° = −2.5 V

7 2HCl → H_2_(g) + Cl_2_(g), Δ*G*° = 200.8 kJ per mole, *E*° = −1.0 V

8 2H_2_O → 2H_2_(g) + O_2_(g), Δ*G*° = 303.9 kJ per mole, *E*° = −0.79 V

As can be observed, despite the presence of Li^+^ in the melt, these cations might not be discharged on the cathode, if the cell potential is restricted smaller than 2.5 V, since the corresponding reactions involving Li^+^ require larger potentials to occur. In other words, the cathodic formation of hydrogen is favorable. It can also be depicted that the evolution of oxygen at the anode is more favorable than that of chlorine. Therefore, at lower cell potentials, hydrogen can solely be discharged on the cathode:

9.1 2H^+^ + 2e → H_2_ on the cathode

9.2 O^2−^ → 1/2O_2_ + 2e on the anode

9 H_2_O_[LiCl]_ → H_2_ + 1/2O_2_, Δ*G*° = 153 kJ, *E*° = −0.79 V, *T* = 650 °C

The formation of hydrogen in molten salts at temperatures around 600–900 °C can provide unique applications as briefly explained in next sections.

## 6. Hydrogen exfoliation of graphite in molten salts

**Fig. 2:**
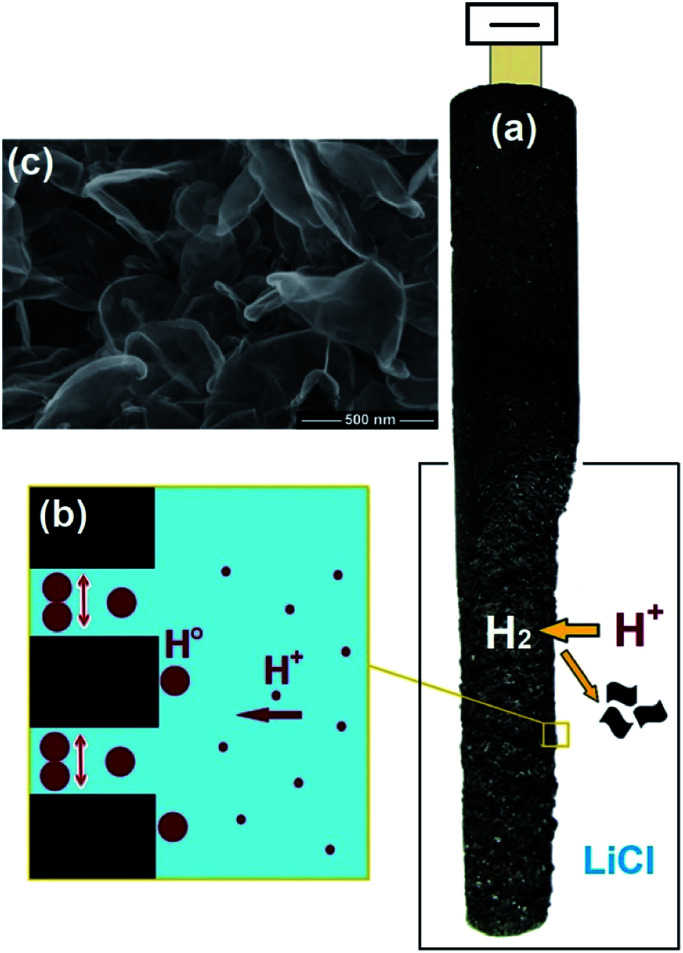
High quality graphene can be produced in molten salts containing hydrogen cations. (a) Protons are neutralised at the surface of cathodically polarised graphite to form hydrogen atoms adsorbed on the graphite surface at 800 °C.The adsorbed hydrogen atoms diffuse into the graphite structure, and then combine to form hydrogen molecules, as identified in (b). The hydrogen gas formed within the graphite structure can exfoliate the material into high quality graphene sheets, as indicated by a typical SEM micrograph in (c).^59,60,71^

The cathodic polarisation of graphite immersed in molten LiCl at 800 °C under a humid atmosphere can lead to the formation of hydrogen on the surface of the graphite, and the hydrogen produced has the capability to exfoliate the graphite material into high quality graphene nanosheets possessing an attractive combination of properties including high electronic conductivity, as high as 5.8 × 10^5^ S m^−1^.^71–75^ The process is briefly illustrated in Fig. 2. The exfoliation process is suggested to occur in three stages. First, atomic hydrogen adsorbed on the graphite surface (
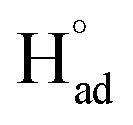
) is formed by the discharge of protons under the cathodic polarisation:10
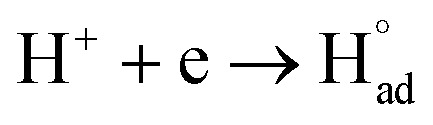


The rapid diffusion of 
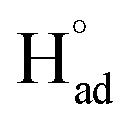
 into the graphite lattice is highly possible, as confirmed by the temperature dependency of the diffusion coefficient of atomic hydrogen into the graphite structure (*D*_H°_, cm^2^ s^−1^):^76^

11 *D*_H°_ = 2.0 × 10^−3^ exp(−6.09 × 10^−20^/*k*_B_*T*), *T* = 300–1700 °C

In this equation, *k*_B_ is the Boltzmann constant (1.38 × 10^−23^ J K^−1^) and *T* is the temperature (K). According to (11), at 25 °C and 800 °C, *D*_H°_ has the values of 9.2 × 10^−10^ and 3.3 × 10^−5^ cm^2^ s^−1^, respectively, expressing a five order of magnitude difference in atomic hydrogen diffusion speed at the higher temperature. Such a diffusion rate allows hydrogen atoms to diffuse deeply into the bulk of graphite,^60,77^ leading to the formation of diffused hydrogen species (
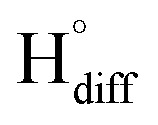
).12
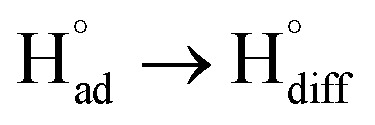


Diffused hydrogen atoms can then combine to form hydrogen molecules within the graphite structure:13
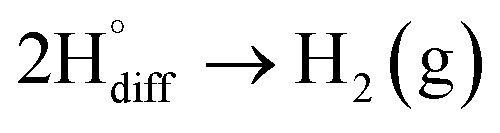


Such hydrogen molecules (with a size of 0.25 nm) created within the hexagonal graphite lattice (with an interlayer spacing of 0.33 nm) have sufficient kinetic energy to exfoliate the graphite material in a green and economic way.^60^ The consumables of the process are electrical energy and water, and no harmful by product is produced, hence the process is environmentally safe. The cost and the specific energy consumption for the preparation of graphene in molten LiCl can be estimated to be low at about US $10–20 and 25 kW h per kilogram of the graphene product, respectively. The molten salt graphene has presented superior performance in various applications including composite anode materials for high performance lithium ion batteries,^78–80^ electrode materials for supercapacitors,^62^ reusable adsorbents,^81,82^ high performance ceramic composites^83^ and precursors for the facile fabrication of nanodiamonds.^84–87^ The application of molten salt produced graphene nanosheets as an efficient reusable adsorbent is based on its high surface area and density of edge sites, as well as its high structural stability, providing the nanosheets with the potential to become recovered after the adsorption of organic contaminants from aqueous solutions by a simple heat treatment process is air.^81^

The crystallinity, and therefore, the electrical conductivity of molten salt produced nanostructured carbon materials have been reported to be high, due to the improving effects brought about by the ionic melt, including the reactive dissolution of impurities from carbonaceous materials into molten salts, the reduction of the *d*-spacing fluctuation between graphitic basal planes, and high diffusion rate of species in the molten salt environment, enhancing the crystallisation of carbon products.^87–92^

## 7. Hydrogen reduction of metal oxides in molten salts

**Fig. 3:**
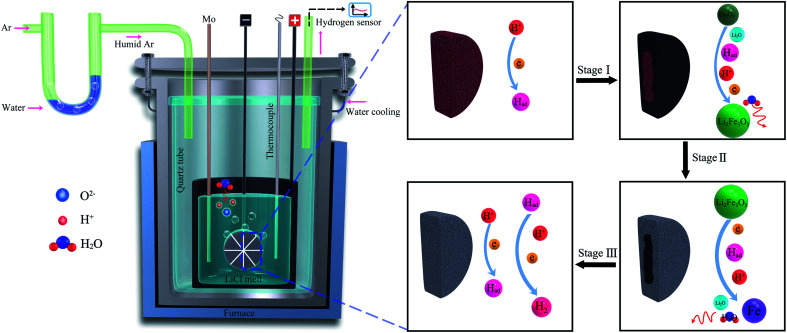
Hydrogen can be produced in molten LiCl under a humid argon atmosphere, and the hydrogen produced can reduce Fe_2_O_3_ to Fe.^93^

The unique advantage of producing hydrogen in molten salt at high temperatures (>600 °C) is that the produced hydrogen can be utilised for the *in situ* reduction of metal oxides. It was demonstrated that hydrogen produced by the dissolution of water in molten LiCl can be used for the *in situ* reduction of iron oxide into metallic iron under a low cell potential of 1–1.4 V in a short period of time (1–5 h) at 660 °C. This approach provides a novel sustainable and economical technology for the carbon-free reduction of iron oxides (Fig. 3). The production yield was found to be high at about 98.4% (1.4 V, 5 h).^93^ In this process, the reduction of protons on the Ni-wrapped Fe_2_O_3_ cathode leads to the electrochemical generation of Li_2_Fe_3_O_5_ at the electrode/melt interface:

14 3Fe_2_O_3_ + 2Li_2_O + H° + H^+^ + e → 2Li_2_Fe_3_O_5_ + H_2_O

Interestingly, it was found that the electrolytic Li_2_Fe_3_O_5_ formed by the reaction (14) has an excellent performance as anode material in lithium ion batteries.^94^ At prolonged electrolysis time, the removal of oxygen from the cathode, and therefore, a complete metallisation of the cathode could be achieved:

15 Li_2_Fe_3_O_5_ + 4H_2_ → 3Fe + Li_2_O + 4H_2_O, Δ*G*° = −1.1 × 10^−16^ kJ mol^−1^, *T* = 660 °C

Whilst the reaction (15) is energetically close to the equilibrium, the presence of a small cathodic potential as well as the dissolution of the reaction products (Li_2_O and H_2_O) into the melt provide further driving forces for the reaction to proceed. At 660 °C, a cell voltage of 0.79 V should be theoretically sufficient for the decomposition of water dissolved in molten salt to form cathodic hydrogen. This result^93^ demonstrated that Fe_2_O_3_ precursor could be reduced to Fe under a low applied voltage of 1.0 V. This achievement can revolutionise the iron production technology, as can be depicted from Table 1.

**Table 1:** A comparison between the molten salt hydrogen reduction of Fe_2_O_3_ with the current commercial iron production methods; including energy consumption (EC, kW h per kg of Fe) and CO_2_ emission (CE, ton per ton of Fe)

Method	T (°C)	EC	CE
Blast furnace^95–98^	1400–2300	3.5–5.0	1.8
Water electrolyser-direct-reduction^98^	800	3.5–5.9	—
H_2_O-molten salt^61,71,93^	660	2.5	—

**Fig. 4:**
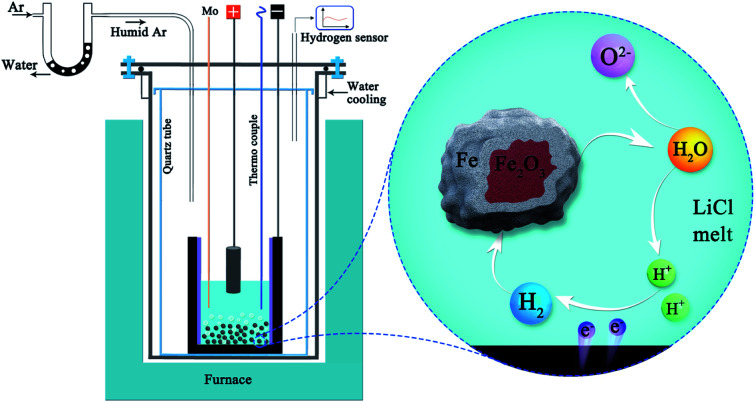
Schematic representation of the molten salt production of hydrogen and its utilisation for direct reduction of Fe_2_O_3_ powders suspended in the melt. Water can repeatedly be decomposed and formed during the process to reduce iron oxides.^61^

Interestingly, individual iron oxide particles simply immersed in molten LiCl at 660 °C can also be reduced to metallic iron particles by the hydrogen gas generated *in situ* in the melt by the electrochemical decomposition of water at a low voltage of only 0.97 V,^61^ as illustrated in Fig. 4, under the influence of hydrogen gas generated on the graphite crucible:

16 Fe_2_O_3_ + 3H_2(electro-generated)_ → 2Fe + 3H_2_O_[LiCl]_, Δ*G*° = −12.5 kJ, *T* = 650 °C

In another work, it was demonstrated that water can repeatedly be decomposed and regenerated in molten LiCl at 680 °C under a low cell voltage of only 1 V, leading to the clean production and the *in situ* consumption of hydrogen to reduce cobalt oxide into highly faceted cobalt microcrystals with a low energy consumption of 1150 kW h per ton of reduced cobalt, providing considerable advantages over the alternative approaches available for cobalt production.^99^

It was explained that the hydrogen gas generated on the cathode (reaction (9.1)) can either escape from the cathode (reaction (17)) or alternatively be consumed for the reduction of cobalt oxide (reaction (18)).

17 H_2_ (melt) → H_2_ (atmosphere)

or

18 4H_2_ + Co_3_O_4_ (pellet) → 3Co + 4[H_2_O]_LiCl_, Δ*G*° = −236.3 kJ at 670 °C

**Fig. 5:**
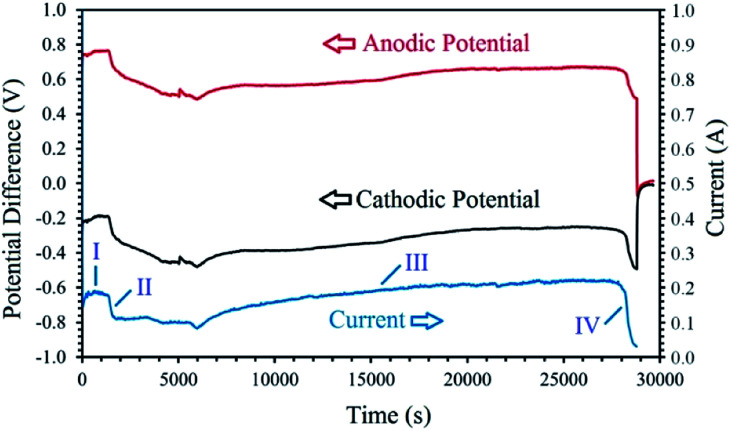
The changes of cell current and cathodic/anodic voltages measured *versus* electrolysis time during the electrolysis of LiCl at 1 V using a cobalt oxide pellet wrapped with Ni wire served as the cathode, and a graphite crucible served as the anode under humid Ar flow. The cathodic and anodic potential differences were measured between the cathode/anode and a Mo pseudo-reference electrode immersed in the melt.^99^

Fig. 5 exhibits the voltage contributions between the graphite anode and the metal oxide cathode, and a Mo pseudo-reference electrode immersed in the melt during the process. As can be observed, although the constant cell voltage of 1 V is applied throughout the process, the cathodic and anodic potentials vary with the electrolysis time. The *I*–*t* and *V*–*t* curves exhibit various distinct stages, based on which the cathodic generation of hydrogen and its utilisation can be explained. The electrolysis initiates with a cathodic and anodic potential of −0.35 V and 0.65 V, respectively, contributing to the high capacitive current observed (this section is not clear in Fig. 5). Then, the values of cathodic and anodic voltages change to around −0.25 V and 0.75 V, respectively, corresponding to the stage I in Fig. 5. During this stage, the cathodic reaction is mainly characterised by the high yield generation of hydrogen, reaction (9.1), on the fresh Ni wire surfaces wrapping the cobalt oxide pellet. After around 30 min, a continuous increase in the cathode potential (and the counterpart reduction of the anodic voltage) is observed, related to the stage II of the process. This stage is associated with the gradual passivation of the Ni wire surfaces, increasing the cathodic impedance, and consequently, the increase of the cathode voltage and the resulting current drop. During the stage I and II, the hydrogen released on the cathode have the opportunity to reduce the surface of the cobalt oxide to form metallic cobalt, proving more active surfaces for the electrochemical production of hydrogen, and hence, leading to a gradual recovery of current and the initiation of stage III. In this stage, the combination of reactions (9) and (18) will maintain the concentration of hydrogen cations adjacent to the cathode, and therefore, an efficient reduction of the oxide phase occurs. Then, at the end of the process, the cathodic voltage sharply increases to the values around −0.5 V due to the passivation of the metallic cobalt cathode, and the shortage of hydrogen cations available adjacent to the cathode. The passivation is mainly due to the formation of hydrogen gas on the cathode with no subsequent metal reduction. Since the experiment was conducted at a low cell voltage of 1 V, the increase in the impedance of the cathode results in a sharp current decay, as observed in the *I*–*t* curve. The mechanism explained above, is responsible for the electrochemical production of hydrogen and subsequent chemical reduction of cobalt oxides by the newly generated hydrogen through different stages.

**Fig. 6:**
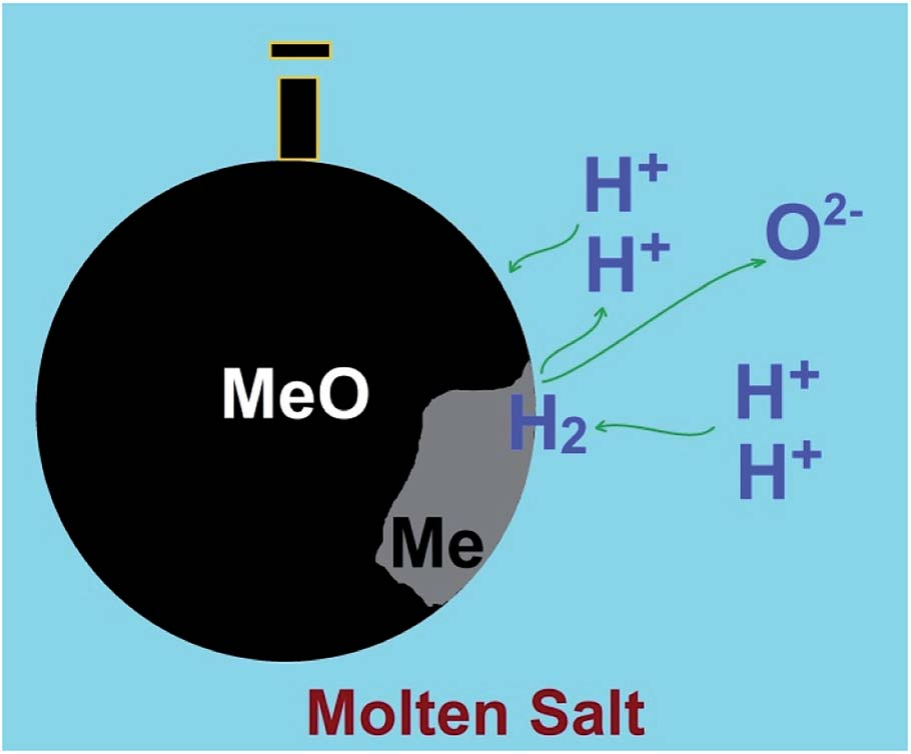
Hydrogen cations present in molten salts can be reduced on cathodically polarised metal oxides to form water and the corresponding metal. The dissolution of produced water in the molten salt leads to the regeneration of hydrogen cations to precede the reduction process.

The low cost and green fabrication of metallic powders using this innovative approach should be interesting, particularly considering the rise of additive manufacturing that utilises metallic powders for the precise, fast and economical manufacturing of components for various industries including aerospace, automotive, energy, and biomedical applications.^100–102^

It is worth mentioning that in the traditional hydrogen reduction processes, metal oxides are reduced by a hydrogen gas stream, from which only a small fraction of hydrogen molecules can contribute to the reduction process^41,42^ and the rest flows away from the reduction zone. In contrast, in the molten salt process, hydrogen is gradually formed in the vicinity of metal oxides, and therefore, a substantially less amounts of hydrogen is required, contributing to the high efficiency of the process.

## 8. Remarks and opportunities

### 8.1. Green production and utilisation of hydrogen in molten salts

The molten salt technology described in the previous sections is able to decompose water at a low cell voltage as low as 1 V to produce hydrogen at the temperature window of 600–900 °C. The hydrogen produced can practically reduce Fe_2_O_3_ added to the molten salt (reaction (16)) without a need for consuming further energy, since its changes of Gibbs free energy is negative (−17 kJ at 700 °C). Moreover, the energy released by the hydrogen reduction of metal oxides provides sufficient energy to maintain the high temperature of the molten salt. Interestingly, the temperature window of 600–900 °C is favourable for the hydrogen reduction of a number of other metal oxides to their corresponding metals/alloys.

I should be mentioned that in the water assisted molten salt reduction of metal oxides, hydrogen cations in the molten salt can be reduced on metal oxide cathodes to form metal and water. The latter is instantly dissolved in the molten salt to re-form hydrogen cations. Therefore, theoretically, a small amount of water in molten salts should be sufficient to reduce a high amount of metal oxides immersed in the melt, at a low cell potential which just high enough to decompose water at high temperatures (<1 V). This mechanism, which is highlighted in Fig. 6, can be applied for a variety of metal oxides.

Table 2 presents a selected number of metal oxides that can thermodynamically be reduced in molten salts under a low cell potential of around 1 V, under the influence of the generated hydrogen. The changes of Gibbs free energy of these reactions are negative, and therefore, can proceed instantly upon the generation of cathodic hydrogen. For the case of oxides such as WO_3_, although the Δ*G*° has a positive value at 700 °C, negative values of Δ*G*° can still be achieved at *T* > 825 °C. Although this temperature can easily be obtained using LiCl (melting point ≈ 605 °C, boiling point ≈ 1380), other molten salts with lower values of vapour pressure, such as CaCl_2_ (melting point ≈ 770 °C, boiling point ≈ 1930 °C) can be more appropriate at higher temperatures. It should be mentioned that, unlike salts such as SnCl_2_,^105^ molten salts like CaCl_2_ (ref. 106 and 107) and ZnCl_2_ (ref. 108) have an obvious capability of being hydrolysed at high temperatures to produce HCl, indicating their potential for being considered appropriate candidates to be included in the molten salt mediums for the production of hydrogen. Further studies should be conducted to elucidate the performance of alternative salt systems.

**Table 2:** Representative reactions that can be conducted by the cathodic hydrogen produced in molten salts at around 1 V, together with their corresponding changes of Gibbs free energy at 700 °C

Hydrogen reduction reaction	Δ*G*° (kJ)
Fe_2_O_3_ + 3H_2_ → 2Fe + 3H_2_O	−16.7
MoO_3_ + 3H_2_ → Mo + 3H_2_O	−83.0
PbO + H_2_ → Pb + H_2_O	−72.3
CuO + H_2_ → Cu + H_2_O	−125.7
SnO_2_ + 2H_2_ → Sn + 2H_2_O	−9.1
CoO + H_2_ → Co + H_2_O	−28.8
NiO + H_2_ → Ni + H_2_O	−43.3
WO_3_ + 3H_2_ → W + 3H_2_O	9.7

It is worth mentioning that the thermal energy required to prepare molten salts can be supplied from waste thermal energy recovered from industrial processes. The wide range of possibilities, shown in Table 2, provides a new horizon in the green production of metals and alloys using water by applying a potential in the range of 1–2.5 V. This includes the green production of stainless steels, Ni-based super alloys, and novel high entropy alloys in powder form. Green production of metal and alloy powders should support the novel manufacturing techniques such as additive manufacturing. The other areas include the reprocessing of nuclear fuels, the recovery of waste metals and alloys, as well as the green and CO_2_-free extraction of metals and alloys from natural ores. Moreover, the intermediate products produced during the molten salt processes, such as Li_2_Fe_3_O_5_ (reaction (14)),^94^ can be interesting for energy applications. Molten salt preparation of metal hydrides should also be highly interesting for applications such as advanced catalysis^103^ and energy storage.^104^

### 8.2. Solar energy powered molten salt production of advanced materials

**Fig. 7:**
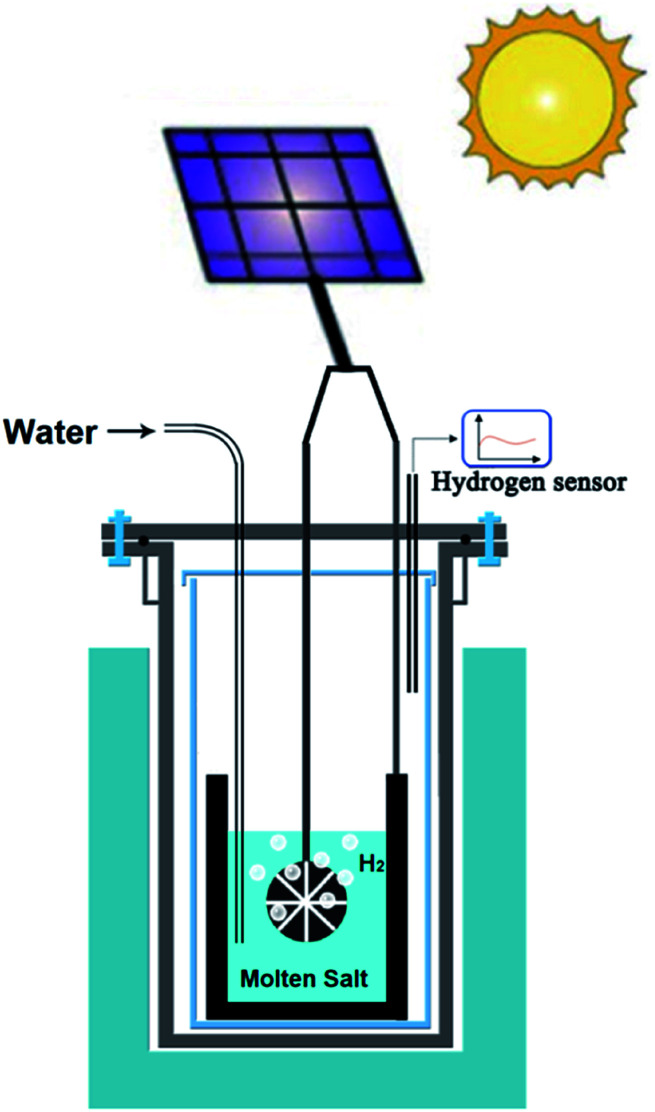
Schematic representation of the solar powdered hydrogen production and utilisation in molten salts.

As an extension of opportunities outlined in the previous section, photovoltaic (PV) technology can be utilised for the production of hydrogen, graphene, advanced alloys and hybrid structures in molten salts. Unlike the case of room temperature electrolysers that require more than 1.8 V, the voltage required to produce hydrogen in molten salts (∼0.9 V) can be supplied by advanced perovskite single-solar cells with potentials in the range of 0.96–1.24 V.^109,110^ This combination can lead to the highly efficient generation of hydrogen by solar energy. Fig. 7 schematically represents the proposed approach.

The characteristics mentioned above are very interesting especially where the large-scale green production of metallic materials is concerned. However, the molten salt approach, like many other high temperature processes, has its own challenges, such as the corrosion of the reactors at high temperatures under the influence of molten salts.^111–114^ Moreover, the level of expertise required to conduct molten salt electrochemical experiments may be higher than those in low temperature operations creating a barrier for researchers to effectively utilise this approach. These limitations can be significantly reduced/eliminated in large-scale molten salt operations, such as the case of Hall–Héroult process for aluminium smelting.

### 8.3. Other considerations and future research

From a mechanism point of view, the electrochemical production and utilisation of hydrogen in high temperature molten salts, discussed in this article, is a new approach based on the dissolution of water (or a hydrogen containing compound) in molten salts, leading to the formation of protons. The electrochemically generated hydrogen formed by the cathodic discharge of such protons at high temperatures can then reduce metal oxides to their corresponding metals/alloys and also produces water as the by product. The latter can be dissolved again in the melt, by which the process proceeds effectively. Therefore, the solubility of water in molten salts is one of the key parameters influencing the process, and in turn depends on various other parameters, including the type of the molten salt system and the temperature.

Despite its interesting nature, however, there is only limited experimental information available in the literature concerning the solubility of hydrogen containing compounds such as water,^115–120^ HCl^121,122^ and HF^123,124^ in molten salts. This is mainly due to the complications involved in such experiments at high temperatures, and also the fact that in traditional molten salt processes, particularly, water has commonly been considered as an undesired impurity that should be eliminated as much as possible from the salt.^125–128^

From the available literature, it is known that the solubility of various gases in high temperature molten salts can be classified into two groups; *i.e.* physical and chemical. The former is represented by the dissolution of nonpolar noble gases in molten metal halides, in which the dissolution can be explained by the creation of a cavity with the same size as the gas molecule in the melt. In this case, the solubility can be correlated by equating the free energy of the gas-melt solution to the free energy of formation of holes considering the surface tension of the melt as the solvent.^129,130^ Such solutes might still exhibit polarisation effects, for instance by the polarisation of the salt ions around the solute particles due to the symmetry breaking created by the presence of such particles.^117^

The solubility of polar water in molten LiNO_3_ and LiNO_3_–KNO_3_ can be as high as 3.5 × 10^−4^ mole of water per mole of the salt. However, the water dissolution process involved was found to be reversible, and based on electrostatic ion–dipole interactions without the occurrence of a hydrolysis reaction.^111^

In contrast to the physical type, the chemical dissolution of water in molten salts is considered technologically important and rather undesirable in various fields such as the electrolytic production of aluminium in cryolite-based melts. It this case, the dissolution process proceeds through the hydrolysis reaction between water and fluoride salts, leading to the formation of hydrolysis products including OH^−^ and H^+^ in the bulk of molten salt. These ionic species can act as charge carriers, influencing the current efficiency of the process. Furthermore, the excessive formation of corrosive HF is of concern.^123,131^ The chemical solubility of water in pure NaF and CaF_2_ was estimated to be as high as 100 and 36 ppm at around 1000 and 1400 °C, respectively.^123^

In chloride molten salts, the eutectic LiCl–KCl is technologically important for applications such as the processing of used nuclear fuels,^132,133^ and therefore, its interaction with water has received some research interest.

Accordingly, it is known that the water uptake of this molten salt system can reach around 0.001 mole of H_2_O per mole of LiCl^134^ at around 400 °C. Also, the solubility of HCl in this eutectic salt has been studied, which is also relevant to our case since protons might be formed by introducing HCl (as the solute) into the molten salt (as the solvent), without the involvement of O^2−^. The protons can be supplied by bubbling of HCl in the melt for several hours.

An interesting discussion can be found in the work of Laitinen and Plambeck^135^ who saturated the purified eutectic LiCl–KCl with HCl, and studied the cell voltage required to form H_2_ and Cl_2_ on Pt electrodes:^2^HCl → H_2_ + Cl_2_, *E*_cell_ = +1.0161 ± 0.0050 V

In another work, Van Norman and Tivers^122^ employed chronopotentiometry to investigate the same system using a glassy carbon working electrode, and determined the apparent diffusion coefficient of protons to be around 2 × 10^−4^ cm^2^ s^−1^, which is an order of magnitude higher than those of most other species in molten salts.

Minh and Welch^136^ studied the cathodic reduction of hydrogen chloride dissolved in the melt by a combination of chronopotentiometry and linear sweep voltammetry using platinum served as the working electrode and also the pseudo-reference electrode, and graphite as the counter electrode. After the saturation of the melt with HCl, a well-defined cathodic reduction wave could be observed in the corresponding voltammogram at −0.55 V *vs.* the Pt pseudo-reference electrode. The peak disappeared when HCl was purged from the eutectic melt with argon. They further found that the reduction reaction proceeds by a reversible one-electron transfer process, and that the diffusion coefficients obtained are considerably high (1.8–2.7 × 10^−4^ cm^2^ s^−1^). The high diffusion coefficients observed could be attributed to the small ionic radius of non-solvated protons in comparison with other ions, providing protons with high mobility within the free volume of the melt rather than its ionic lattice. Moreover, the activation energy for inter-diffusion of protons was estimated to be low at 3.7 kJ mol^−1^, which is an order of magnitude lower than those of alkali metal ions.^136^

Future investigations should be designed to more specifically evaluate key issues influencing the molten salt generation of hydrogen such as the selection of molten salt and electrodes materials, as well as the processing conditions including the temperature and cell voltage. Electrochemical measurements should also provide useful information about the mechanisms and kinetics of hydrogen evolution in more details. Finally, the generated hydrogen should provide an opportunity to investigate the green production of a range of metals and alloys.^137^ This research is ongoing in my laboratory.

## Conflicts of interest

There are no conflicts to declare.

## Supplementary Material
